# ^Downregulation of microRNA-9-5p promotes synaptic remodeling in the chronic phase after traumatic brain injury^

**DOI:** 10.1038/s41419-020-03329-5

**Published:** 2021-01-05

**Authors:** Jingchuan Wu, Hui Li, Junchi He, Xiaocui Tian, Shuilian Luo, Jiankang Li, Wei Li, Jianjun Zhong, Hongrong Zhang, Zhijian Huang, Xiaochuan Sun, Tao Jiang

**Affiliations:** 1 Department of Neurosurgery, The First Affiliated Hospital of Chongqing Medical University, Chongqing, 400016 China; 2 Department of Neurosurgery, General Hospital of The YangTze River Shipping, Wuhan Brain Hospital, Wuhan, Hubei 430014 China; 3 College of Pharmacy, Chongqing Key Laboratory of Biochemistry and Molecular Pharmacology, Chongqing Medical University, Chongqing, Yuzhong 400016 China; 4 Department of Ultrasound, Zhongnan Hospital of WuHan University, Wuhan, 430071 China; 5 Dept of Computer Science, City University of Hong Kong, 83 Tat Chee Ave, Kowloon Hong Kong, China; 6 BGI Education Center, University of Chinese Academy of Sciences, Shenzhen, 518083 China; 7 Department of Neurosurgery, Beijing TianTan Hospital, Capital Medical University, Beijing, 100050 China; 8 Beijing Neurosurgical Institute, Capital Medical University, Beijing, 100050 China; 9 China National Clinical Research Center for Neurological diseases, Beijing, China

**Keywords:** Synaptic plasticity, Trauma

## Abstract

The level of microRNA-9-5p (miRNA-9-5p) in brain tissues is significantly changed in the chronic phase after traumatic brain injury (TBI). However, the effect of miRNA-9-5p on brain function after TBI has not been elucidated. In this study, we used a controlled cortical impact (CCI) model to induce TBI in Sprague–Dawley rats. Brain microvascular endothelial cells (BMECs), astrocytes, and neurons were extracted from immature Sprague–Dawley rats and cocultured to reconstruct the neurovascular unit (NVU) in vitro. The results showed that downregulation of miRNA-9-5p in the chronic phase contributed to neurological function recovery by promoting astrocyte proliferation and increasing the release of astrocyte-derived neurotrophic factors around injured brain tissues after TBI. A dual-luciferase reporter assay validated that miRNA-9-5p was a post-transcriptional modulator of thrombospondin 2 (Thbs-2), and downregulation of miRNA-9-5p promoted Thbs-2 expression in astrocytes. Furthermore, we verified that Thbs-2 can promote Notch pathway activation by directly binding to Jagged and Notch. Through in vitro experiments, we found that the expression of synaptic proteins and the number of synaptic bodies were increased in neurons in the NVU, which was constructed using astrocytes pretreated with miRNA-9-5p inhibitor. Moreover, we also found that downregulation of miRNA-9-5p promoted Thbs-2 expression in astrocytes, which activated the Notch/cylindromatosis/transforming growth factor-β-activated kinase 1 pathway in neurons and promoted the expression of synaptic proteins, including post-synaptic density protein 95 and synaptotagmin. Based on these results, miRNA-9-5p may be a new promising prognostic marker and treatment target for TBI.

## Introduction

Traumatic brain injury (TBI) is one of the leading causes of disability all over the world^1^. The period of post-TBI can be divided into three phases: the acute phase is the first 7 days after TBI, the subacute phase is between 1 and 3 weeks after TBI, and the chronic phase begins 3 weeks after TBI^2–5^. Excessive inflammation and blood–brain barrier (BBB) destruction in the acute phase and subacute phase of TBI aggravate cerebral edema, resulting in ischemia and hypoxia in local brain tissues and inducing brain cell apoptosis^6,7^. At present, the clinical treatments for severe TBI (sTBI) mainly focus on the acute stage after brain injury. Most clinical treatments aim to reduce cranial pressure and alleviate the inflammatory response in the acute stage after TBI, which can maintain the blood and oxygen supply to injured brain tissues^8–10^. However, nearly 30% patients who survive an sTBI still have severe neurological deficits^11^. Although a large number of studies have shown that stem cell therapy^12^ and hyperbaric oxygen therapy^13,14^ in the acute phase can promote neurological function recovery after TBI, few therapeutic methods can be applied in actual clinical treatment for chronic convalescence after TBI.

The neurovascular unit (NVU) is mainly composed of the BBB and neurons^15^, and a stable NVU is the basis of normal brain function. Therefore, repair of the NVU is crucial for neurological function recovery in the convalescence phase after TBI. In recent years, many researchers have reported that non-coding RNAs play an important role in maintaining stable and normal NVU function. MicroRNAs (miRNAs) are short (19–28 nucleotides) endogenous non-coding RNAs that regulate protein synthesis at the post-transcriptional level^16^. Previous studies have verified that the levels of various miRNAs are significantly changed in brain tissues after TBI, which can restore the normal function of the NVU by inhibiting the apoptosis of neurons and reducing the destruction of the BBB. The level of miR-21-5p increases in the acute phase after TBI, and upregulation of miR-21-5p can promote neurological function recovery by reducing BBB damage and alleviating the neuroinflammatory response^17^. PTEN, which is an important regulatory protein of the AKT/mTOR pathway, is a direct target of miR-23a-3p. Previous studies have reported that upregulation of miR-23a-3p can inhibit cortical neuron apoptosis and improve neurological function by regulating the AKT/mTOR pathway^18^. miRNA-9 is highly expressed in the central nervous system and plays an important role in the differentiation and synaptic formation of neural stem cells^19^. Our previous studies have shown that continuous upregulation of miRNA-9 in the acute phase after TBI can inhibit the apoptosis of neurons, alleviate the inflammatory response, reduce BBB destruction, and promote neurological function recovery^20^. However, whether continuous upregulation of miRNA-9 only in the chronic convalescence phase can also promote neurological function recovery after TBI remains unknown.

In this study, we established a controlled cortical impact (CCI) model in rats and an NVU model in vitro to investigate the effects of miRNA-9 in the chronic recovery phase after TBI. The results may provide a new treatment target for TBI.

## Materials and methods

### Animals

Adult male (~16 weeks, weighing 250–300 g) and neonatal Sprague–Dawley rats were purchased from the Experimental Animal Center of Chongqing Medical University (Chongqing, China) and SLAC laboratory animal corporation (Shanghai, China). The adult rats were raised in a temperature-controlled room (22 ± 2 °C) with a 12-h light/12-h dark cycle and provided with free access to food and water. All adult rats were identified by earmarks. Microsoft Excel 365 (Microsoft Corporation, USA) was used to generate a table of random numbers, and a number was assigned to each rat for simple random grouping. The random number was sorted from small to large and assigned to each group. There were 450 adult and 37 immature Sprague–Dawley rats were required for this study. Forty-seven rats that died in 24 h post CCI were excluded. All animal procedures were conducted in compliance with the Animal Research: Reporting In Vivo Experiments (ARRIVE) guidelines and the National Institutes of Health Guide for the Care and Use of Laboratory Animals. All animal experiments were evaluated and approved by the Chongqing Medical University Animal Experimentation Committee and the ethics committee of Yangtze River Shipping General Hospital. The detailed information about the animal distribution and usage was listed in Supplementary Table 1.

### CCI model and intracerebroventricular injection

The CCI model was used to simulate moderate TBI in rats using a controlled impactor device (TBI-0310 TBI Model System; Precision Systems and Instrumentation, Chongqing, China). All rats were anesthetized by intraperitoneal injection of 10% chloral hydrate (3 mL/kg) (Sigma, USA) and sufentanil (50 μg/kg) (Sigma) and maintained at 36–37 °C after anesthesia induction. The impact parameters were set as a velocity of 3.5 m/s, a depth of 2.5 mm, and a dwell time of 200 ms. A 4.0-mm-diameter craniotomy was performed on the right parietal bone 4.0 mm lateral to the sagittal suture and 5.0 mm caudal to the bregma. After the impact, thrombin (500 U/mL) (Solarbio, Beijing, China) was locally perfused on the wound to stop the bleeding. The bone window was closed with bone wax (Johnson & Johnson, USA), and the wound was sutured.

The miRNA-9-5p agomir and antagomir (RiboBio, Guangzhou, China) were used to upregulate and downregulate the level of miRNA-9-5p in vivo, respectively. The agomir and antagomir were diluted with sterilized phosphate-buffered saline (PBS) solution to 0.1 and 0.2 mM, respectively, according to the manufacturer’s instructions and previous research^21,22^. A Hamilton brain infusion syringe was stereotaxically inserted into the contralateral ventricle through a bone hole (coordinates: 1.5 mm caudal to the bregma; 1.1 mm lateral to the midline; 4.5 mm deep from the surface of the skull). The mixture of oligomers (5 µL) was continuously injected into the contralateral ventricle over 5 min. The speed of insertion and withdrawal was controlled at 1.5 mm/min. All rats were divided into a whole-process treatment group and a chronic treatment group according to different study times. Due to metabolic consumption of agomir and antagomir, the miRNA oligomers were injected twice at 15 min and 14 days after the CCI in the whole-process treatment group (double injection (DI) group), while the miRNA oligomers were injected only once at 14 days after the CCI in the chronic treatment group (single injection (SI) group). Some measures were taken to reduce the rats’ suffering after the impact procedure. Ibuprofen (50 mg/kg) (Solarbio) was administered orally for 3 days to alleviate pain, and penicillin sodium (15 mg/kg) (Solarbio) was injected intraperitoneally once a day for 7 days to prevent infection. All rats were returned to their cages and provided with free access to food and water. The rats in the sham group underwent the same surgical procedure, including anesthesia, craniotomy, intracerebroventricular injection with sterilized PBS, and scalp suture. Brain tissues with a neurological severity score diameter of 8 mm from the impacted area were collected for subsequent testing.

### Modified neurological severity score

Modified neurological severity score (mNSS), which reflects motor function, sensory function, reflexes, and balance, was used as previously reported to evaluate the effects of miRNA oligomers on TBI^21^. The rats were examined before the CCI and 1, 3, 7, 14, 21, 28, and 35 days after the CCI. mNSS data were collected and analyzed by a person who was blinded to the animal group assignments to avoid any bias from the observer.

### Morris water maze test

The spatial learning and memory of the rats were evaluated using the Morris water maze test^23,24^. Each rat was released into the water tank from quadrants 1 to 4 and allowed to search for the platform in 90 s, which was placed 5 cm under the water surface. The test was ended when the rat either found and remained on the platform for 5 s or did not find the platform within 90 s. If the rat could not find the platform, it was guided to the platform and kept there for 20 s. All tests were performed twice a day from 30 to 34 days after the CCI. Then, each rat was released into quadrant 2, and the time required for the rat to find the platform, which was in quadrant 4, was recorded at 35 days after the CCI. After 8 h, the platform was removed, and each rat was released into the tank again. The swimming path of all rats in the water tank was recorded. We recorded the distances that the rats swam, the times that the rats passed through the platform, and the times that the rats spent in quadrant 4 to study the effects of different interventions on TBI. The data of Morris water maze test were collected and analyzed by a person who was blinded to the animal group assignments to avoid any bias from the observer.

### Cell culture

Brain microvascular endothelial cells (BMECs) were extracted from 1-week-old newborn rats as described in our previous study^25^. The newborn rats were anesthetized by inhalation of 4% isoflurane for 2 min. The cerebral cortex was collected and separated into 1-mm^3^ pieces, and then the pieces were digested with 0.1% collagenase type-2 (Thermo Fisher, Shanghai, China) and DNase I (39 U/mL) (Thermo Fisher) at 37 °C for 30 min. The mixtures were centrifuged at 1000 r.p.m. for 8 min. The precipitates were collected and resuspended in 20% bovine serum albumin. The mixtures were centrifuged at 2000 r.p.m. for 10 min again. The precipitates were digested with collagenase/dispase (1 mg/mL) and DNase I (39 U/mL) for 1 h at 37 °C and centrifuged at 1000 r.p.m. for 8 min. Next, the cell clusters were maintained in Dulbecco’s modified Eagle’s medium/nutrient mixture F-12 (DMEM/F-12) supplemented with 15% fetal bovine serum (FBS). Cell culture plates were placed in a 37 °C incubator with 5% CO_2_, and the cell medium was changed every 3 days.

Astrocytes were extracted from 1-day-old rats^25^. The newborn rats were also anesthetized by inhalation of 4% isoflurane for 2 min. The brains were collected, and the gray matter was separated into 1-mm^3^ pieces, which were minced and digested with 0.1% collagenase type-2 (Thermo Fisher Scientific) and DNase I (Thermo Fisher Scientific) (39 U/mL) at 37 °C for 30 min. The mixtures were centrifuged at 1000 rpm for 8 min. After centrifugation, the precipitates were resuspended in DMEM/F-12 medium with 10% FBS and 1% glutamine (2 mM) (Thermo Fisher Scientific). When the rate of cell fusion reached 80%, the cells were shaken at 250 rpm for 18 h to purify the astrocytes. The astrocytes were maintained in DMEM/F-12 with 10% FBS and placed in a 37 °C incubator with 5% CO_2_, and the medium was changed every 3 days.

Neurons were extracted from fetal rats after gestation for 20 days as previously described^25^. Gray matter was minced and digested with 0.1% collagenase type-2 and DNase I (39 U/mL). After centrifugation, the precipitates were resuspended in DMEM/F-12 containing 10% FBS and 1% glutamine (2 mM). The mixtures were seeded in plates precoated with 0.01% poly-l-lysine. The neurons were maintained in neurobasal medium supplemented with 2% B27 and 1% glutamine (2 mM). After 3 days, the cells were treated with cytarabine (Selleck, Shanghai, China) (5.0 mg/mL) to inhibit non-neuronal cell growth.

### miRNA mimic and inhibitor transfection in vitro

The miRNA-9-5p mimic (RiboBio) and inhibitor (RiboBio) were used to upregulate and downregulate the level of miRNA-9-5p in vitro, respectively. The concentrations of the mimic and inhibitor were 50 and 100 nM in the cell culture medium, respectively. The method was performed according to the following instructions.

### Thrombospondin 2 siRNA transfection in vitro

We used thrombospondin 2 (Thbs-2) small interfering RNA (siRNA) (RiboBio) to regulate the expression of Thbs-2 in astrocytes. The transfection was performed when the fusion rate of astrocytes reached 80% in a 24-well cell culture plate according to the manufacturer’s instructions. All transfected cells were placed in a CO_2_ incubator at 37 °C for 24 h and collected for the study.

### Reconstruction of the NVU in vitro

The NVU was reconstructed as in a previous study^25^. Astrocytes (5 × 10^5^ cells/cm^2^) were plated on poly-l-lysine-coated inserts (polyethylene terephthalate membranes, 1.0 μm, Millipore, Germany) and incubated for 5 h. Then, the inserts were placed in matching wells and cultured for 24 h. BMECs (5.0 × 10^5^ cells/cm^2^) were plated on the inner side of the insert membrane, which was coated with the Matrigel matrix. After 3 days, the insert was placed in another matching well containing neurons (1 × 10^5^ cells/cm^2^) that had already been cultured for 5 days. BMECs, astrocytes, and neurons were cocultured to establish the NVU model in vitro, while BMECs and astrocytes were cocultured to establish a BBB model in vitro. According to the different pretreatments applied to astrocytes, the NVU was divided into the ACM (astrocyte co-cultured model) group, ACM (inhibitor) group, and ACM (siRNA + inhibitor) group. The control group included only BMECs.

### Prediction of target genes and the dual-luciferase reporter assay

The TargetScan, miRWalk, miRTarBase, and miRDB databases were used to predict candidate target genes of miRNA-9-5p. HEK293T cells (1 × 10^5^) (ATCC) passaged a maximum of eight times were cultured in 24-well plates. The cells were transfected with Thbs-2-3′-untranslated region (UTR)-wild-type (WT) or Thbs-2-3′-UTR-mutant (mt). The miRNA-9-5p mimic or miRNA-9-5p-NC was transfected into HEK293 cells with riboFect^TM^ CP reagent. Luciferase activity was measured 24 h after transfection with the Dual Luciferase Reporter Assay System (Promega, Madison, USA). The results were normalized to Renilla activity. All the experimental steps were performed according to the manufacturer’s instructions.

### RNA extraction and real-time quantitative PCR

Total RNA was extracted from the injured brain tissue using TRIzol reagent (Thermo Fisher Scientific) according to the manufacturer’s instruction. The concentration of RNA was tested using the Nanodrop spectrophotometer (NanoDrop 2000, Thermo Fisher Scientific, USA), and the integrity was assessed by gel electrophoresis. The total RNA was reverse-transcribed into complementary DNA (cDNA), and the cDNA was mixed with the specific Bulge-Loop^TM^ miRNA Primer (RiboBio) and miDETECT A Track miRNA qRT-PCR Starter Kit Reagents (RiboBio). U6 was used as an endogenous control for normalization. Real-time quantitative PCR was performed using the ABI PRISM 7500 Real-Time PCR instrument (Applied Biosystems, Chongqing, China). All steps were performed according to the manufacturers’ instructions. The changing level of miRNA-9-5p was calculated using the comparative threshold cycle (Ct) method and 2^−ΔΔCt^ as previously described^20^.

### Immunoblotting and co-immunoprecipitation-IP

Brain tissues and cells were homogenized with RIPA lysis buffer (Beyotime, China) containing protease and phosphatase inhibitors. The debris was eliminated by centrifugation at 12,000 r.p.m. at 4 °C for 15 min. The protein concentration in the supernatant was quantified using a Bicinchoninic Acid Protein Assay Kit (Beyotime, China). Then, the supernatants were mixed with 5× sodium dodecyl sulfate loading buffer (Beyotime, China) and boiled at 100 °C for 5 min. Immunoblotting was performed as previously described^17^. Glyceraldehyde 3-phosphate dehydrogenase was used as the endogenous control. The antibodies are shown in Supplementary Table 2.

Briefly, the co-immunoprecipitation-IP was performed using Protein A/G agarose (Beyotime, China) following the manufacturer’s instructions^26^. Normal rabbit anti-immunoglobulin (IgG) (Abcam, UK) was used as a control antibody. The mixing samples containing input (protein without pretreatment), IgG (protein pretreated with A/G agarose and rabbit anti-IgG), anti-Jagged protein pretreated with A/G agarose, or anti-Notch protein pretreated with A/G agarose. The antibodies are shown in Supplementary Table 3. The immunoreactive bands were visualized using an Enhanced Chemiluminescence Kit (Bio-Rad, Shanghai, China), and the band densities were quantified and analyzed using the Fusion-FX7 System (Vilber Lourmat, France).

### Immunofluorescence staining

Briefly, the immunofluorescence staining was performed as previously reported^20^. The tissue slices or cells were treated with the primary antibody overnight at 4 °C and then incubated with secondary antibody for 1 h at room temperature. The nucleus was stained with 4′,6-diamidino-2-phenylindole (Thermo Fisher Scientific). The antibodies are shown in Supplementary Table 4. All images were captured with a fluorescence microscope (Eclipse Ti-S, Nikon, Japan). The mean fluorescence intensity was recorded and analyzed.

### Statistical analysis

All statistical analyses were performed with the SPSS software 19.0, and statistical figures were created using GraphPad Prism 8.00 software. The values are expressed as the mean ± standard deviation (SD). No outliers were excluded from the analysis. Grubbs’ test was used to identify outliers among the analyzed data. The Shapiro–Wilk test was applied to test for normality, and the data were normally distributed. Student’s *t* test was used to compare two groups. Comparisons among multiple groups were performed by one- or two-way analysis of variance using Tukey’s honestly significant difference post hoc test and Holm–Bonferroni correction. Differences were considered statistically significant at *P* < 0.05.

## Results

### Alteration of miRNA-9-5p affected neurological outcomes after CCI

**Fig. 1:**
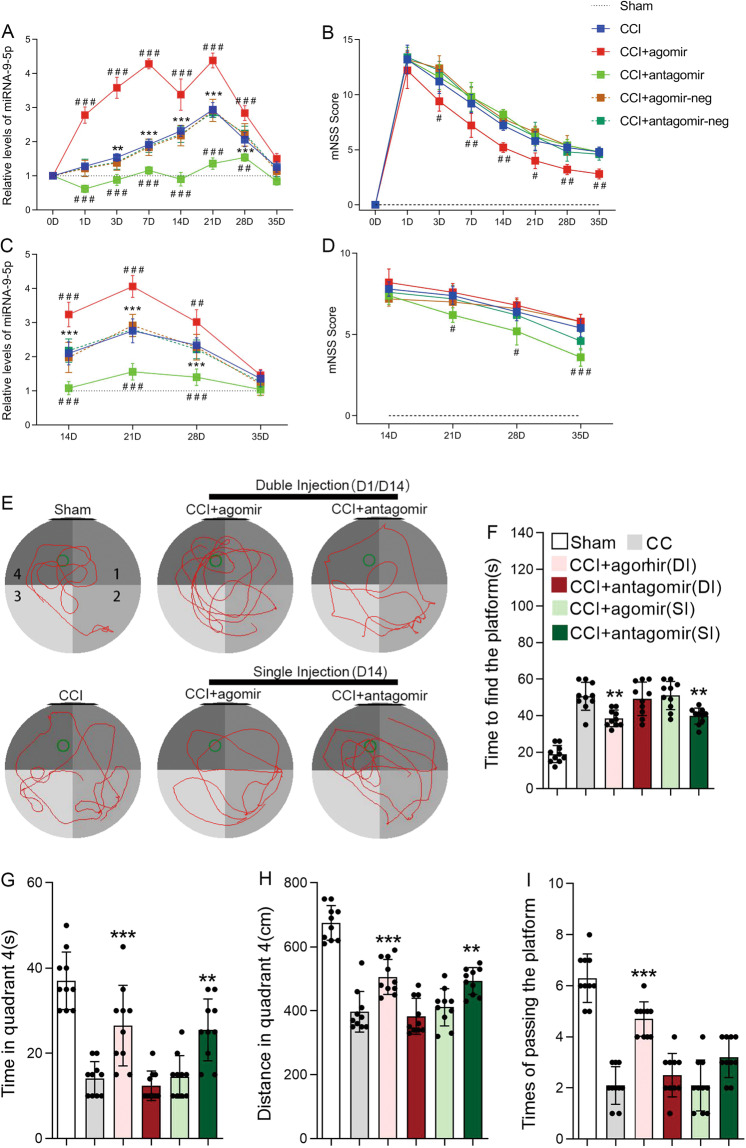
Alteration of miRNA-9-5p affected neurological outcomes after CCI. **A** The temporal profile of the miRNA-9-5p level in traumatic foci from 1 to 35 days was detected by qRT-PCR. **B** The neurological outcome after treatment with miRNA-9-5p oligomers from 1 to 35 days was evaluated by the mNSS. **C** The temporal profile of the miRNA-9-5p level in traumatic foci from 14 to 35 days was detected by qRT-PCR. **D** The neurological outcome after treatment with miRNA-9-5p oligomers from 14 to 35 days was evaluated by the mNSS (*n* = 5/group). (**P < 0.01, ***P < 0.001 versus the sham group; ^#^*P* < 0.05, ^##^*P* < 0.01, ^###^*P* < 0.001 versus the CCI group.) **E** Spatial learning and memory were evaluated using the Morris water maze test. **F** The time to finding the platform. **G** The times to passing through the platform. **H** The distance traversed in quadrant 4. **I** The time spent in quadrant 4 (*n* = 10/group) (*P < 0.05, **P < 0.01, ***P < 0.001 versus the CCI group) (CCI: control cortex impact, DI: double injection, SI: single injection). Error bars indicate mean ± SD.

We induced controlled cortical injury in rats to detect the effects of miRNA-9-5p on neurological outcomes after CCI. The results showed that the level of miRNA-9-5p was continuously increased and peaked at 21 days after TBI (Fig. 1A). Compared with the CCI group, staged injection of agomir or antagomir at 1 and 14 days after the CCI resulted in a sustained significant increase or decrease in miRNA-9-5p in injured brain tissues (Fig. 1A). Neurological outcomes were evaluated using the mNSS (Fig. 1B), and the results showed that the mNSS in the CCI + agomir group was decreased compared with that in the CCI group (Fig. 1B). The spatial learning and memory of rats were evaluated using the Morris water maze test (Fig. 1E), and we found that compared to the rats in the CCI group, the rats in the CCI + agomir group required less time to find the platform (Fig. 1F), spent more time (Fig. 1G), and traversed longer distances in quadrant 4 (Fig. 1H), and more frequently passed through the platform point (Fig. 1I). These results are partly consistent with those from our previous research^20^. Unexpectedly, however, we found that when the miRNA-9-5p oligomers were injected only at 14 days after CCI, although the level of miRNA-9-5p in injured brain tissues was similar to that observed with DI (Fig. 1C), the mNSS and Morris water maze test results were different. The results showed that compared to that in the CCI group, the mNSS in the CCI + antagomir group was decreased (Fig. 1D), and the rats performed better in the Morris water maze test, requiring less time to find the platform (Fig. 1F) and spending more time (Fig. 1G) and traversing longer distances in quadrant 4 (Fig. 1H).

### Downregulation of miRNA-9-5p promoted the proliferation of astrocytes and the release of astrocyte-derived neurotrophic factors in the chronic phase after TBI

**Fig. 2:**
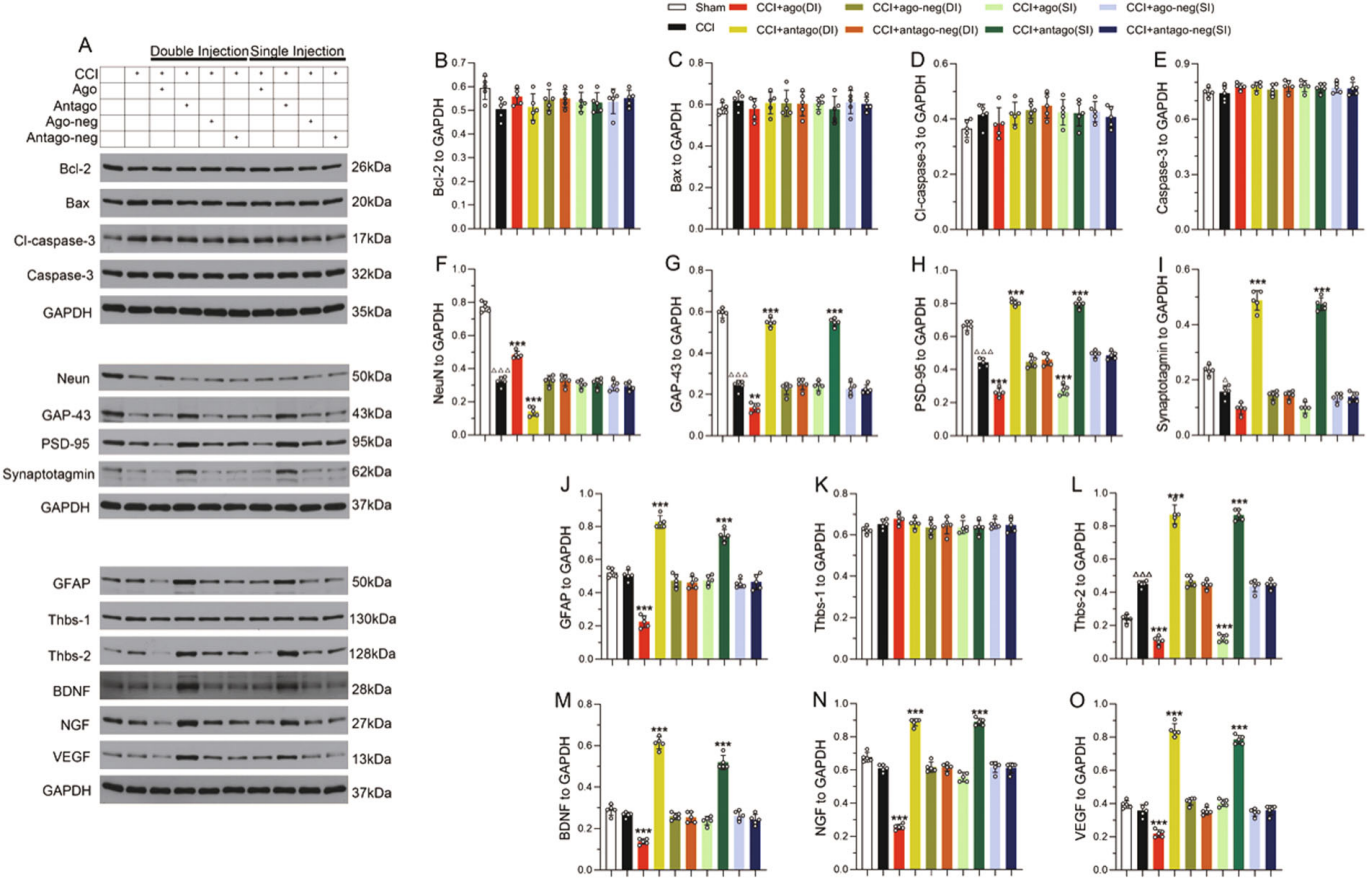
Downregulation of miRNA-9-5p promoted the proliferation of astrocytes and the expression of astrocyte-derived neurotrophic factors in the chronic phase after TBI. **A** The immunoblotting and quantitative data of **B** Bcl-2, **C** Bax, **D** cleaved caspase-3, **E** caspase-3, **F** NeuN, **G** GAP-43, **H** PSD-95, **I** synaptotagmin, **J** GFAP, **K** Thbs-1, **L** Thbs-2, **M** BDNF, **N** NGF, and **O** VEGF were detected at 35 days after CCI (*n* = 5/group). (^△^*P* < 0.05, ^△△△^*P* < 0.001 versus the sham group; **P < 0.01, ***P < 0.001 versus the CCI group) (CCI: control cortex impact, DI: double injection, SI: single injection). Error bars indicate mean ± SD.

To further investigate the effects of miRNA-9-5p oligomers in different phases after CCI, we detected apoptosis-related molecules (Fig. 2A) in traumatic foci at 35 days. The results showed that the expression of apoptotic proteins, including Bcl-2 (Fig. 2B), Bax (Fig. 2C), cleaved caspase-3 (Fig. 2D), and caspase-3 (Fig. 2E), was not obviously changed in either the DI group or the SI group compared to the sham group. Next, we detected some neural molecular markers and found that sustained upregulation of miRNA-9-5p after CCI increased the expression of NeuN in traumatic foci after CCI, while regulating the level of miRNA-9-5p only in the chronic phase post injury had no effect on the expression of NeuN (Fig. 2F). The results also showed that the miRNA-9-5p antagomir promoted the expression of GAP-43 (Fig. 2G), post-synaptic density protein 95 (PSD-95) (Fig. 2H), and synaptotagmin (Fig. 2I) in both the DI group and the SI group. In addition, we found that continuous upregulation of miRNA-9-5p inhibited the expression of glial fibrillary acidic protein (Fig. 2J) and some astrocyte-derived neurotrophic factors, including Thbs-2 (Fig. 2L), brain-derived neurotrophic factor (BDNF) (Fig. 2M), nerve growth factor (NGF) (Fig. 2N), and Vascular endothelial growth factor (VEGF) (Fig. 2O), in the DI group after CCI, while downregulation of miRNA-9-5p promoted the expression of Thbs-2 (Fig. 2L), BDNF (Fig. 2M), NGF (Fig. 2N), and VEGF (Fig. 2O) in both the DI group and the SI group. Statistically significant differences in Thbs-1 expression were not observed (Fig. 2K) in any groups.

### miRNA-9-5p targeted the 3′-UTR of Thbs-2 mRNA, and downregulation of miRNA-9-5p promoted the expression of Thbs-2 in astrocytes

**Fig. 3:**
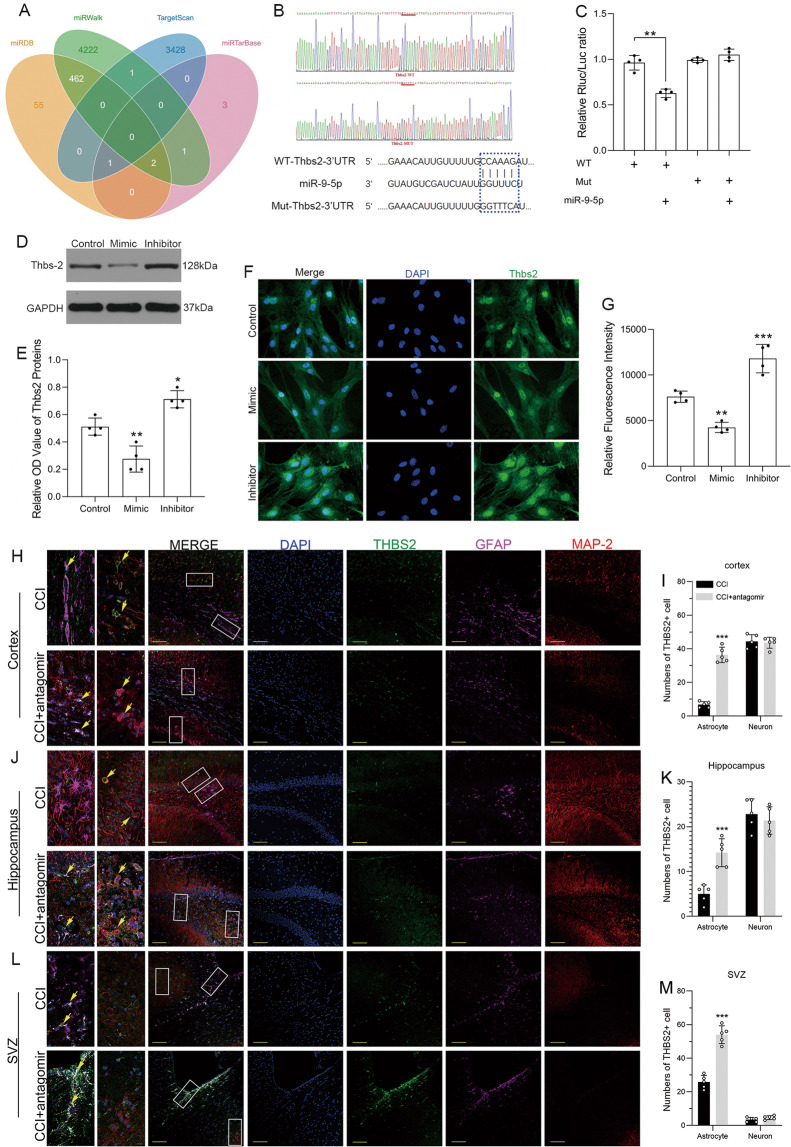
miRNA-9-5p targeted the 3′UTR of Thbs-2 mRNA, and downregulation of miRNA-9-5p promoted the expression of Thbs-2 in astrocytes. **A** Prediction of the miRNA-9-5p target and **B** construction of the Thbs-2 mutation site. **C** The dual luciferase reporter assay (*P < 0.05, **P < 0.01 versus the WT group). **D** The immunoblotting and **E** quantitative data of Thbs-2 in astrocytes. **F** The immunofluorescence (bar = 100 μm) and **G** quantitative data of Thbs-2 in astrocytes (*n* = 4/group) (*P < 0.05, **P < 0.01, ***P < 0.001 versus the control group). **H** The immunofluorescence (bar = 100 μm) and **I** quantitative data of Thbs-2 in the cortex. **J** The immunofluorescence (bar = 100 μm) and **K** quantitative data of Thbs-2 in the hippocampus. **L** The immunofluorescence (bar = 100 μm) and **M** quantitative data of Thbs-2 in the SVZ (*n* = 5/group) (*P < 0.05, **P < 0.01, ***P < 0.001 versus the CCI group) (WT: wild type, Mut: mutant type, CCI: control cortex impact, SVZ: subventricular zone). Error bars indicate mean ± SD.

We used bioinformatics databases to explore the potential target of miRNA-9-5p (Fig. 3A) and found that Thbs-2 may be the direct target of miRNA-9-5p. We constructed mutant vectors (Fig. 3B) and confirmed that miRNA-9-5p targeted the 3′-UTR of Thbs-2 mRNA using a luciferase reporter assay. The miRNA-9-5p mimic decreased luciferase activity in the WT group (Fig. 3C). In addition, the results of immunoblotting (Fig. 3D, E) and immunofluorescence (Fig. 3F, G) also showed that compared to the control group, the miRNA-9-5p mimic decreased the expression of Thbs-2, while the miRNA-9-5p inhibitor increased the expression of Thbs-2 in astrocytes in vitro. Next, we verified this result in vivo again. The immunofluorescence results showed that downregulation of miRNA-9-5p increased the number of Thbs-2-positive astrocytes in the cerebral cortex (Fig. 3H, I), hippocampus (Fig. 3J, K), and subventricular zone (SVZ) (Fig. 3L, M) in the chronic phase after brain injury, but inhibited the expression of Thbs-2 in neurons in the cerebral cortex and hippocampus.

### Downregulation of miRNA-9-5p in astrocytes promoted neuron synaptic remodeling in the NVU in vitro

**Fig. 4:**
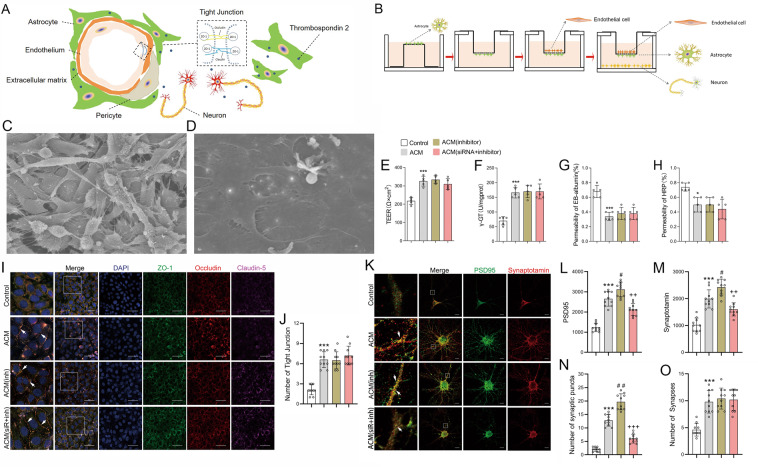
The effect of miRNA-9-5p inhibitor on BBB stability and synaptogenesis in vitro. **A** Schematic of the NVU. **B** The procedure used to reconstruct the NVU in vitro. **C** BMECs and **D** astrocytes visualized by scanning electron microscopy (bar = 30 μm). Quantitative data of the effects of the miRNA-9-5p inhibitor on **E** TEER, **F** γ-GT activity, and **G** EB and **H** HRP permeability in vitro (*n* = 5/group). **I** The immunofluorescence (bar = 50 μm) and **J** quantitative data of the tight junction structure in vitro. **K** The immunofluorescence (bar = 10 μm) and quantitative data of **L** PSD-95 and **M** synaptotagmin in neurons. The numbers of **N** synaptic puncta and **O** synapses of neurons (*n* = 10/group) (*P < 0.05, ***P < 0.001 versus the control group; ^#^*P* < 0.05, ^##^*P* < 0.01 versus the ACM group; ^++^*P* < 0.01, ^+++^*P* < 0.001 versus the ACM (inhibitor) group) (ACM: astrocyte co-cultured model, inh: inhibitor, siR: siRNA). Error bars indicate mean ± SD.

We used preconditioned astrocytes to investigate the effect of miRNA-9-5p on the NVU in vitro. An NVU model establishment diagram is shown in Figs. 4A, B. First, astrocytes and BMECs were cocultured to establish the BBB model in vitro. Then, neurons and the BBB model were cocultured again to establish the NVU model in vitro. The in vitro BBB consisted of a continuous single layer of BMECs (Fig. 4C) and astrocytes (Fig. 4D). The stability of the BBB was evaluated by measuring transepithelial electrical resistance (TEER), γ-GT activity, and permeability to Evans blue (EB) and horseradish peroxidase (HRP). The results showed that the TEER values (Fig. 4E) and γ-GT activity (Fig. 4F) in the ACM group were significantly increased compared to those in the control group, which consisted of only BMECs, while the permeability to EB (Fig. 4G) and HRP (Fig. 4H) was significantly decreased in the ACM group compared to the control group. However, no statistically significant differences in TEER values, γ-GT activity, or permeability to EB and HRP were found among the ACM group, ACM (inhibitor) group, and ACM (siRNA + inhibitor) group. Tight junction (TJ) structures between adjacent BMECs were detected by immunofluorescence staining, which was identified by colocalization of ZO-1, occludin, and claudin-5. The results showed that the TJ number was also increased in the ACM group compared to the control group (Fig. 4I, J), while no significant differences were identified among the ACM group, ACM (inhibitor) group, and ACM (siRNA + inhibitor) group. We also detected the effect of miRNA-9-5p on the synaptic function of neurons in the NVU in vitro. Synaptic puncta, which were identified by colocalization of PSD-95 and synaptotagmin, are the basis of synaptic function for neurons. Compared to that in the control group, the expression of PSD-95 (Fig. 4K, L) and synaptotagmin (Fig. 4K, M) in the ACM group was increased, and the numbers of synaptic puncta (Fig. 4K, N) and neuronal synapses (Fig. 4K–O) in the ACM group were also increased. In addition, we found that downregulation of miRNA-9-5p in astrocytes by the inhibitor promoted the expression of PSD-95 (Fig. 4K, L) and synaptotagmin (Fig. 4K–M) and increased the number of synaptic puncta (Fig. 4K–N) compared to the ACM group. However, silencing Thbs-2 by siRNA can partly reverse the effect of miRNA-9-5p (Fig. 4K–N). The results also showed that regulating the levels of miRNA-9-5p and Thbs-2 in astrocytes did not affect the number of neuronal synapses in the NVU (Fig. 4K–O).

### Downregulation of miRNA-9-5p activated the Notch pathway by promoting the expression of Thbs-2

**Fig. 5:**
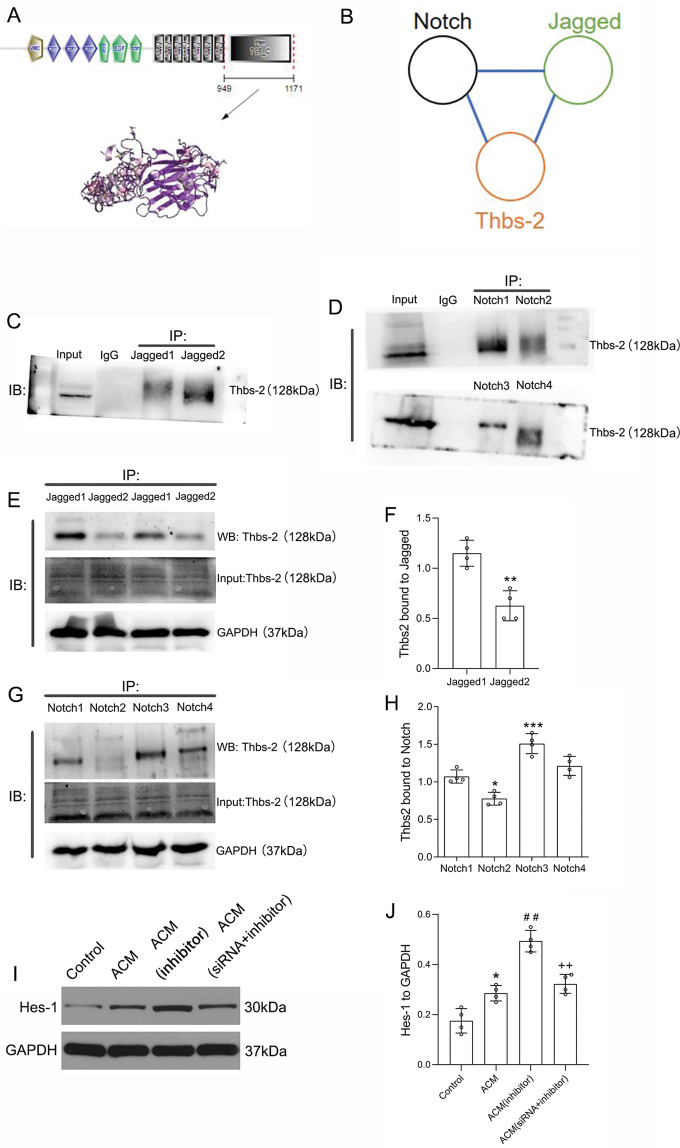
Downregulation of miRNA-9-5p activated the Notch pathway by promoting the expression of Thbs-2. **A** The structure of Thbs-2 protein. **B** Schematic diagram of the relationship among Thbs-2, Notch, and Jagged. **C** Immunoblotting showing the relationship between Thbs-2 and Jagged. **D** Immunoblotting showing the relationship between Thbs-2 and Notch. **E** The immunoblotting and **F** quantitative data of Thbs-2 bound to Jagged1 and Jagged2 (*n* = 4/group) (*P < 0.05, **P < 0.01 versus the Jagged1 group). **G** The immunoblotting and **H** quantitative data of Thbs-2 bound to Notch1, Notch2, Notch3, and Notch4 (*n* = 4/group) (*P < 0.05, **P < 0.01, ***P < 0.001 versus the Notch1 group). **I** The immunoblotting and **J** quantitative data of Hes-1 in neurons (*n* = 4/group) (*P < 0.05 versus the control group; ^##^*P* < 0.01 versus the ACM group; ^++^*P* < 0.01 versus the ACM (inhibitor) group) (ACM: astrocyte co-cultured model). Error bars indicate mean ± SD.

To determine how astrocytes affect neuronal development through the miRNA-9-5p/Thbs-2 pathway, we explored the proteins that interact with Thbs-2 using the String database and found that Thbs-2 may physically interact with both Notch1 and Jagged1 (Fig. 5A, B). We verified this prediction in neurons and confirmed that Thbs-2 directly interacted with Jagged1–2 (Fig. 5C) and Notch1–4 (Fig. 5D). The results also showed that Thbs-2 mainly interacted with Jagged1 (Fig. 5E, F) and Notch3 (Fig. 5G, H). Through in vitro experiments, we found that the expression of Hes-1 was increased in neurons cocultured with astrocytes (Fig. 5I, J). Moreover, the results showed that downregulation of miRNA-9-5p in astrocytes promoted the expression of Hes-1 in neurons in the NVU in vitro (Fig. 5I, J). However, this effect of the miRNA-9-5p inhibitor can be reversed by silencing Thbs-2 (Fig. 5I, J). These data indicated that miRNA-9-5p can promote activation of the Notch pathway in neurons in the NVU by regulating Thbs-2 in astrocytes.

### Downregulation of miRNA-9-5p in astrocytes activated the Notch/CYLD/TAK1 pathway of neurons in the NVU in vitro

**Fig. 6:**
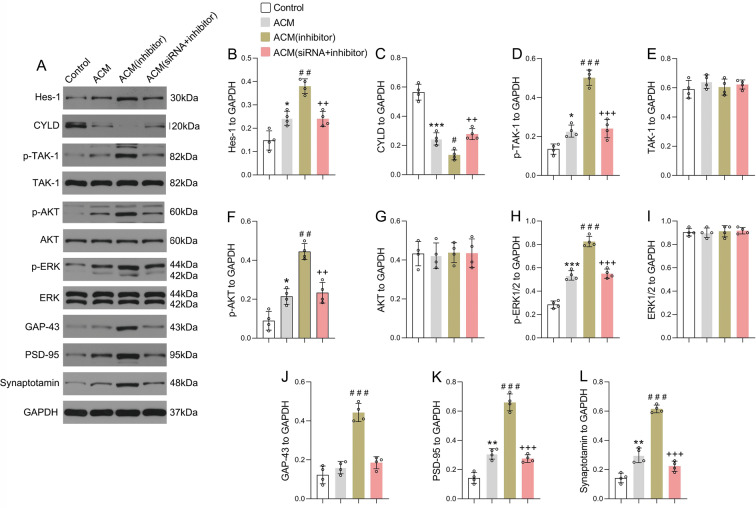
Downregulation of miRNA-9-5p in astrocytes activated the Notch/CYLD/TAK1 pathway of neurons in the NVU in vitro. **A** The immunoblotting and quantitative data of **B** Hes-1, **C** CYLD, **D** p-TAK1, **E** TAK1, **F** p-AKT, **G** AKT, **H** p-ERK, **I** ERK, **J** GAP-43, **K** PSD-95, and **L** synaptotagmin in neurons (*n* = 4/group) (*P < 0.05, **P < 0.01, ***P < 0.001 versus the control group; ^#^*P* < 0.05, ^##^*P* < 0.01, ^###^*P* < 0.001 versus the ACM group; ^++^*P* < 0.01, ^+++^*P* < 0.001 versus the ACM (inhibitor) group) (ACM: astrocyte co-cultured model). Error bars indicate mean ± SD.

**Fig. 7:**
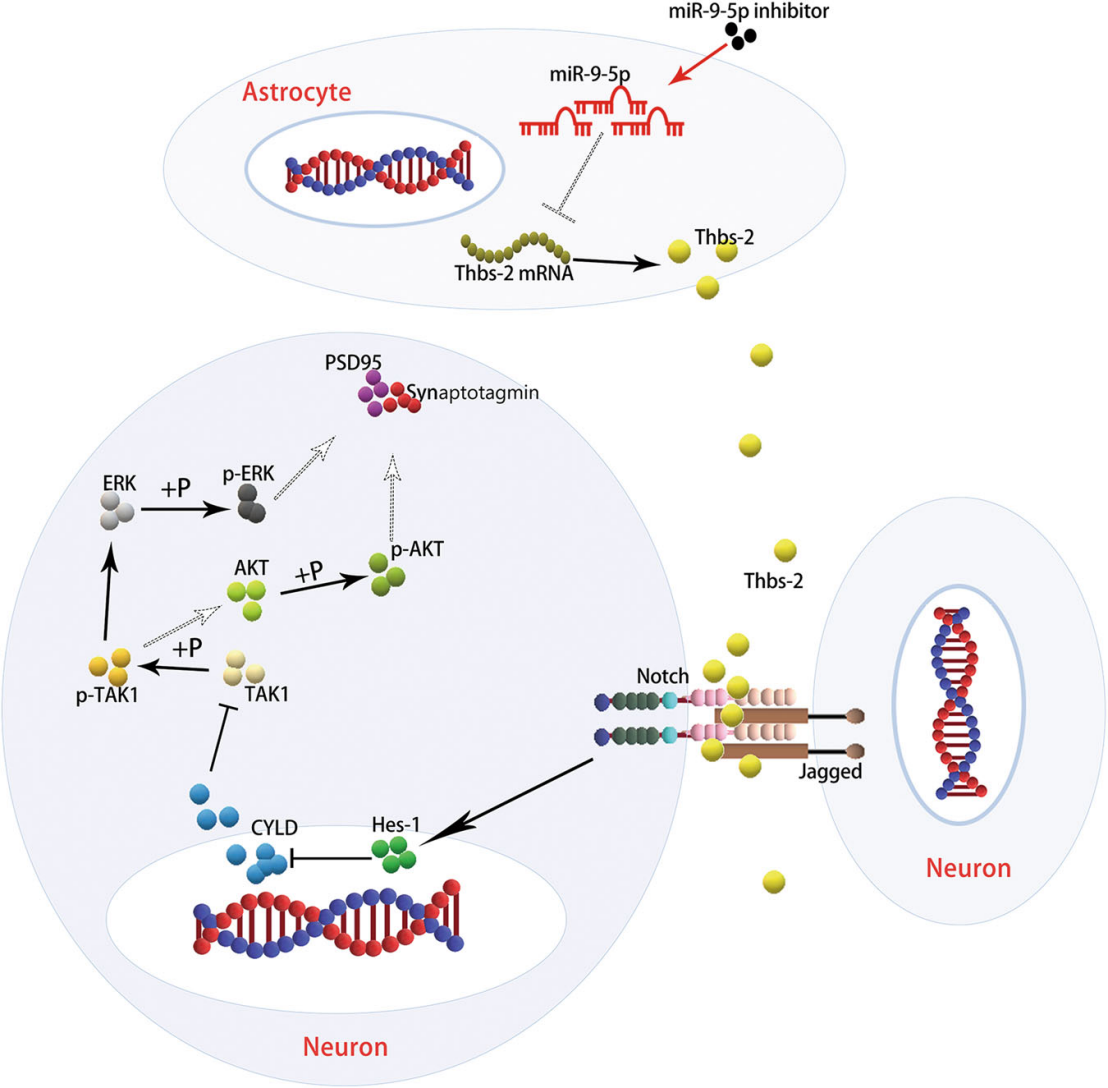
Schematic summary of the miRNA-9-5p/Thbs-2/Notch pathway. The neurovascular unit (NVU) is reconstructed by brain microvascular endothelial cell, astrocyte, and neuron. Transfection of microRNA-9-5p (miRNA-9-5p) inhibitor blocks the inhibitory effect of miRNA-9-5p on thrombospondin 2 (Thbs-2), which promotes the expression of Thbs-2 protein in astrocytes and increases the concentration of Thbs-2 protein between neurons. Moreover, Thbs-2 promotes the activation of Notch pathway for neuron, which blocks the inhibitory effect of cylindromatosis (CYLD) on transforming growth factor-β-activated kinase 1 (TAK1) and promotes the phosphorylation of TAK1 (p-TAK1). The p-TAK1 promotes the phosphorylation of extracellular signal-regulated kinase (ERK) and protein kinase B (AKT), and activates the ERK and AKT pathways, which promotes the expression of neuronal synapse protein including post-synaptic density protein 95 (PSD-95) and synaptotagmin.

To further reveal the mechanism of astrocytes influencing neuronal synapse development through the miRNA-9-5p/Thbs-2 pathway, the cylindromatosis/transforming growth factor-β-activated kinase 1 (CYLD/TAK1) pathway was detected in neurons in the NVU (Fig. 6A). We found that compared to those in the control group, the expression of Hes-1 (Fig. 6B) and p-TAK1 (Fig. 6D) in the ACM group was increased in neurons, while CYLD expression (Fig. 6C) was decreased. The results also showed that the expression of p-AKT (Fig. 6F), p-ERK (Fig. 6H), PSD-95 (Fig. 6K), and synaptotagmin (Fig. 6L) was significantly increased in the ACM group. Moreover, compared to the ACM group, downregulation of miRNA-9-5p in astrocytes promoted the expression of Hes-1 (Fig. 6B) and p-TAK1 (Fig. 6D) in neurons in the NVU while inhibiting the expression of CYLD (Fig. 6C). In addition, compared to that in the ACM group, the expression of p-AKT (Fig. 6F), p-ERK (Fig. 6H), GAP-43 (Fig. 6J), PSD-95 (Fig. 6K), and synaptotagmin (Fig. 6L) was also significantly increased in the ACM (inhibitor) group. However, the activation of the CYLD/TAK1 pathway by the miRNA-9-5p inhibitor can be reversed by inhibiting Thbs-2. The expression of TAK1 (Fig. 6E), AKT (Fig. 6G), and ERK (Fig. 6I) showed no significant change in each group. These data suggested that miRNA-9-5p promoted synapse development by activating the Notch/CYLD/TAK1 pathway in neurons in the NVU through regulation of the expression of Thbs-2 in astrocytes. The proposed mechanism was shown in the schematic representation (Fig. 7).

## Discussion

Extensive clinical data show that the mortality rate of sTBI is extremely high^27^. Even if the patient survives, he often suffers from memory loss, limb hemiplegia, and other severe neurological dysfunction^28^. At present, the clinical treatments for sTBI are limited, and most basic research mainly focuses on the acute phase after TBI. Few effective treatments and basic studies in the chronic stage after TBI are available. However, a large number of patients with neurological deficits in the chronic convalescence phase after TBI require rehabilitation treatment. In recent years, increasing evidence has indicated that miRNAs play an important role in the pathological process after TBI^29–31^. miRNAs have been found to be able to freely pass through the BBB to the peripheral blood via exosomes, providing a basis for miRNA as a biomarker for brain function recovery after TBI^32–34^. Therefore, clarifying the role of miRNAs after TBI may be helpful for formulating treatment strategies in the future. Our previous studies also verified that upregulation of miR-9-5p in the acute phase after TBI can reduce the inflammatory response and BBB destruction, thus promoting neurological function recovery^20^. However, whether the upregulation of miR-9-5p has the same neuroprotective role in the chronic phase after TBI remains unknown.

In this study, we verified that continuous upregulation of miRNA-9-5p after TBI contributed to neurological function recovery. Unexpectedly, however, we found that upregulation of miRNA-9-5p only in the chronic phase after TBI did not promote brain function recovery, while downregulation of miRNA-9-5p in the chronic phase contributed to the recovery of memory and limb function in rats. We examined locally damaged brain tissues and found that downregulation of miRNA-9-5p could promote astrocyte proliferation and the secretion of various glial neurotrophic factors while also promoting the expression of growth-associated protein and synaptic protein in neurons. The NVU, which mainly consists of neurons, astrocytes, and BMECs, is the important structural basis for normal brain function. Previous studies have verified that astrocytes play an important role in tissue repair after brain injury^35,36^, and that restoration of the structure and function of NVUs is beneficial to brain function recovery after TBI^37,38^. Therefore, we speculated that downregulation of miRNA-9-5p in the chronic phase after TBI promoted neurological function recovery by facilitating the functional recovery of NVUs.

According to the mechanism of miRNA, we confirmed that Thbs-2 is one of the downstream target genes for miRNA-9-5p, and that miRNA-9-5p can directly regulate the expression of Thbs-2. Thbs, one category of numerous ECM proteins, regulate inflammation and remodeling after tissue damage^39^. Thbs are virtually undetectable under normal conditions in adult organs, but are secreted by several types of cells in the wound healing process. Thbs-2 is mainly secreted by astrocytes in the nervous system and can promote neuronal synapse formation^40,41^. To verify the effect of the miRNA-9-5p/Thbs-2 axis in astrocytes, we established a multicellular NVU model in vitro. The results showed that astrocytes pretreated with the mRNA inhibitor promoted the formation of synapse puncta in NVUs, but had no influence on the permeability of the BBB. When Thbs-2 was silenced, the ability of miRNA-9-5p inhibitor-pretreated astrocytes to promote synapse puncta formation was significantly weakened. Previous studies have shown that promoting synaptic remodeling is beneficial to the recovery of neurological function after TBI^42,43^. These results are consistent with our findings.

To further explore the mechanism of Thbs-2 in synaptic remodeling in NVU, we analyzed the protein structure of Thbs-2 and found that Thbs-2 can activate the Notch pathway in neurons by directly binding to the proteins Jagged and Notch. Previous studies have reported that the Notch/Hes-1 pathway repressed the expression of CYLD^44,45^, a deubiquitination protease, and that CYLD can regulate the activation of TAK1^46,47^, which is a member of the mitogen-activated protein kinase kinase family. Next, we confirmed that miRNA-9-5p/Thbs-2-induced activation of the Notch pathway can inhibit the expression of CYLD and increase the phosphorylation of TAK1 in neurons, thus promoting activation of ERK and AKT and the expression of synapse proteins.

Some limitations exist in this study. First, the number of experimental rats was limited, which may have caused experimental errors. Second, we reconstructed the NVU in vitro by coculturing BMECs, astrocytes, and neurons. However, recent studies have shown that the complete NVU includes microglia, pericytes, and other cells^48^. Therefore, the reconstructed in vitro NVU did not completely simulate the in vivo NVU. miRNA-9-5p may play a neuroprotective role by regulating other cells in the NVU. In addition, previous studies have confirmed that upregulation of miR-9 can terminate the proliferation of neural stem cells and promote the differentiation of neural stem cells into neurons^49,50^. Other studies have also verified that overexpression of Thbs-4, which is one of the members of the Thb family, can promote the differentiation of neural stem cells into astrocytes, and promote tissue repair after brain injury^51^. In this study, we also found that downregulation of miRNA-9-5p promoted the proliferation of Thbs-2-positive astrocytes in the SVZ. These results suggested that the miRNA-9-5p/Thbs-2 axis may play an important role by influencing the proliferation and differentiation of neural stem cells, but the specific mechanism requires further exploration.

In conclusion, our findings confirmed that the effects of miR-9-5p vary in different phases after TBI. In the chronic phase after TBI, downregulation of miRNA-9-5p promoted the expression of Thbs-2 in astrocytes, which can activate the Notch/CYLD/TAK1 pathway of neurons in the NVU and promote synapse remodeling and neurological function recovery. Overall, this study expands our understanding of miRNA-9-5p and suggests that dynamic monitoring and regulation of miRNA-9-5p levels may be a new treatment direction for TBI.

## Supplementary information

supplement table

Supplementary Figure legend

supplement figure 1

supplement figure 2

supplement figure 3

supplement figure 4

supplement figure 5

supplement figure 6
