# Correction: Downregulation of microRNA-9-5p promotes synaptic remodeling in the chronic phase after traumatic brain injury

**DOI:** 10.1038/s41419-021-03429-w

**Published:** 2021-03-03

**Authors:** Jingchuan Wu, Hui Li, Junchi He, Xiaocui Tian, Shuilian Luo, Jiankang Li, Wei Li, Jianjun Zhong, Hongrong Zhang, Zhijian Huang, Xiaochuan Sun, Tao Jiang

**Affiliations:** 1 Department of Neurosurgery, The First Affiliated Hospital of Chongqing Medical University, Chongqing, 400016 China; 2 Department of Neurosurgery, General Hospital of the YangTze River Shipping, Wuhan Brain Hospital, Wuhan, Hubei 430014 China; 3 College of Pharmacy, Chongqing Key Laboratory of Biochemistry and Molecular Pharmacology, Chongqing Medical University, Chongqing, Yuzhong 400016 China; 4 Department of Ultrasound, Zhongnan Hospital of Wuhan University, Wuhan, 430071 China; 5 Dept of Computer Science, City University of Hong Kong, 83 Tat Chee Ave, Kowloon, Hong Kong, China; 6 BGI Education Center, University of Chinese Academy of Sciences, Shenzhen, 518083 China; 7 Department of Neurosurgery, Beijing TianTan Hospital, Capital Medical University, Beijing, 100050 China; 8 Beijing Neurosurgical Institute, Capital Medical University, Beijing, 100050 China; 9 China National Clinical Research Center for Neurological diseases, Beijing, China

**Keywords:** Synaptic plasticity, Trauma

Correction to: *Cell Death & Disease*

10.1038/s41419-020-03329-5 published online 5 January 2021

The original version of this article unfortunately contained a mistake in Fig. 2. The ‘F’ and ‘G’ graphs in Fig. 2 were in reverse order. The correct figure can be found below. The authors apologize for the mistake. The original article has been corrected.

**Figure:**
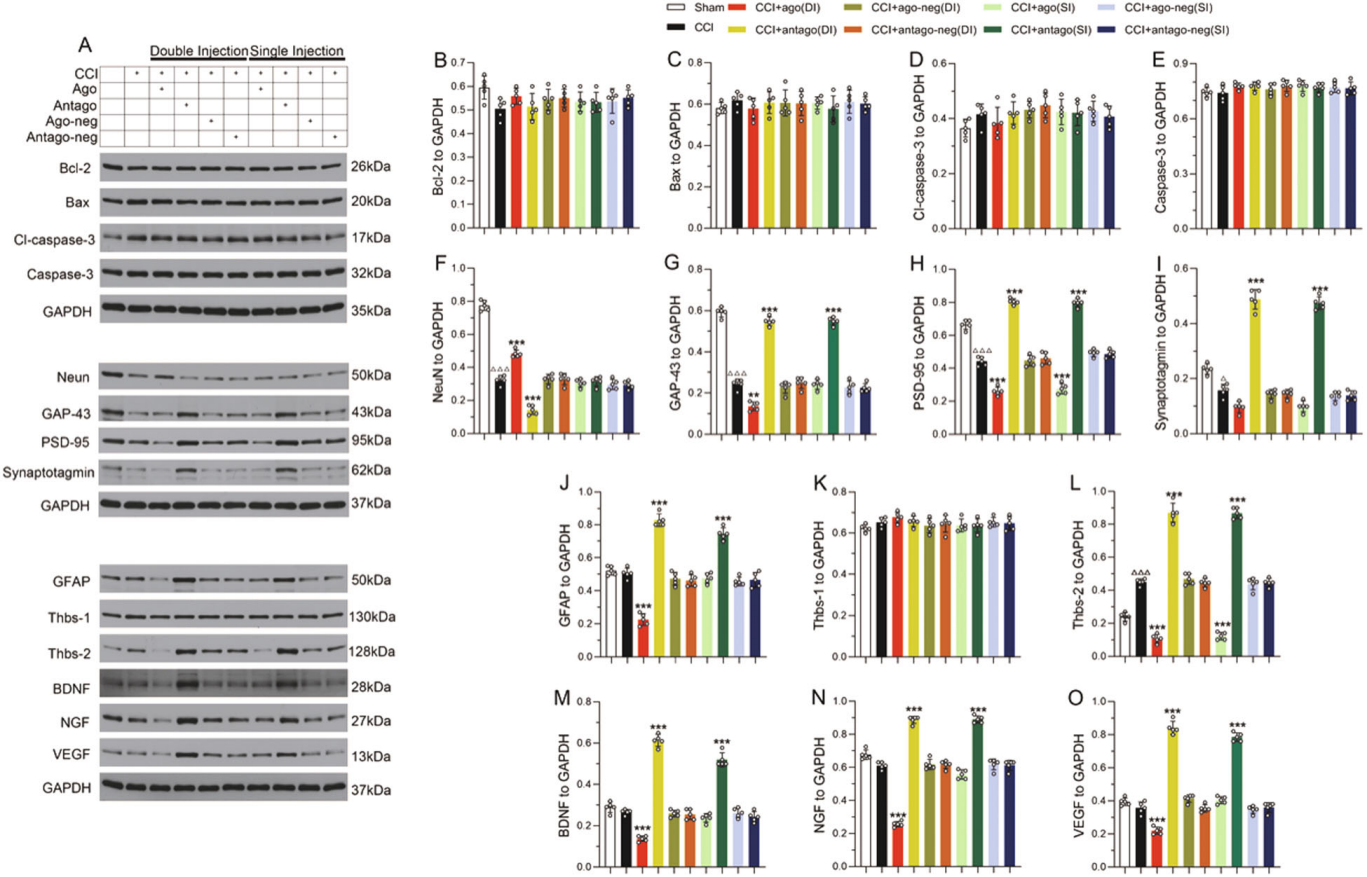
Fig. 2.

