# Supplementary material for: Downregulation of microRNA-9-5p promotes synaptic remodeling in the chronic phase after traumatic brain injury
Source: Cell Death Dis. 2021 Jan 5;12(1):9. doi: 10.1038/s41419-020-03329-5 (PMC7790831; doi:10.1038/s41419-020-03329-5)
Supplement: Supplementary file 1 — supplement table [file 41419_2020_3329_MOESM1_ESM.docx]

**Supplementary Table 1. Animal distribution and usage in this study.**

| Experiments Name | Groups | | PCR  WB  IF  mNSS | MWM | Cell  culture | Death | Mortality |
| --- | --- | --- | --- | --- | --- | --- | --- |
| Experiment1 | Sham | | 5 | - | - | 0 | 0 |
|  | CCI | | 40 | - | - | 4 | 10% |
|  | DI and SI | CCI+agomir | 40x2 | - | - | 7 | 8.75% |
|  |  | CCI+agomir-neg | 40x2 | - | - | 8 | 10% |
|  |  | CCI+antagomir | 40x2 |  |  | 8 | 10% |
|  |  | CCI+antagomir-neg | 40x2 |  |  | 9 | 11.25 |
| Experiment2 | Sham | | - | 10 | - | 0 | 0 |
|  | CCI | | - | 15 | - | 2 | 13.33% |
|  | DI and SI | CCI+agomir | - | 15x2 | - | 5 | 16.67% |
|  |  | CCI+antagomir | -- | 15x2 | -- | 4 | 13.33% |
|  | BMEC | | - | - | 10 | - | - |
| Experiment 3 | Astrocyte | | - | - | 12 | - | - |
|  | Neuron | | - | - | 15 | - | - |
| Total number | Adult:450 | | 365 | 85 | - | 47 | 10.44% |
|  | immature:37 | | - | - | 37 | - | - |

CCI: control cortex impact, PCR: Polymerase Chain Reaction, WB: western blot, IF: Immunofluorescence, mNSS: Modified neurological severity score, MWM: Morris water maze test, DI: double injection, SI: single injection, BMEC: brain microvascular endothelial cell

**Supplementary Table 2. Antibodies used for Immunoblotting in this study.**

| **Name** | **Company** | **Catalog Number** | **Concentration** |
| --- | --- | --- | --- |
| Bcl-2 | Abcam | #ab196495 | 1:1000 |
| Bax | Cell Signaling Technology | #2772 | 1:1000 |
| Cleaved Caspase-3 | Abcam | #ab49822 | 1:1000 |
| Caspase-3 | Abcam | #ab44976 | 1:1000 |
| NeuN | Abcam | #ab128886 | 1:1000 |
| GAP-43 | Proteintech | #16971-1-AP | 1:1000 |
| PSD-95 | Cell Signaling Technology | #3450 | 1:1000 |
| Synaptotagmin | Abcam | #ab13259 | 1:1000 |
| GFAP | Cell Signaling Technology | #80788 | 1:1000 |
| Thbs-1 | Abcam | #ab1823 | 1:1000 |
| Thbs-2 | Abcam | #ab112543 | 1:1000 |
| BDNF | Abcam | #ab226843 | 1:1000 |
| NGF | Abcam | #ab52918 | 1:1000 |
| VEGF | Abcam | #ab46154 | 1:1000 |
| Hes-1 | Novus | #OTI4H1 | 1:1000 |
| CYLD | Abcam | #ab60266 |  |
| p-TAK-1 | Cell Signaling Technology | #9339 | 1:1000 |
| TAK-1 | Cell Signaling Technology | #4505 | 1:1000 |
| p-AKT | Cell Signaling Technology | #4060 | 1:1000 |
| AKT | Cell Signaling Technology | #9272 | 1:1000 |
| p-ERK | Cell Signaling Technology | #4370 | 1:1000 |
| ERK | Cell Signaling Technology | #4695 | 1:1000 |
| GAPDH | Proteintech | #10494-1-AP | 1:1000 |
| HRP-conjugated Goat Anti-Mouse IgG | Proteintech | #SA00001-1 | 1:3000 |
| HRP-conjugated Goat Anti-Rabbit IgG | Proteintech | # SA00001-15 | 1:3000 |

**Supplementary Table 3. Antibodies used for CO-IP in this study.**

| **Name** | **Company** | **Catalog Number** | **Concentration** |
| --- | --- | --- | --- |
| Jagged-1 | Invitrogen™ | #J.841.5 | 1:50 |
| Jagged-2 | Cell Signaling Technology | #C23D2 | 1:50 |
| Notch-1 | Cell Signaling Technology | #D1E11 | 1:50 |
| Notch-2 | Cell Signaling Technology | #D76A6 | 1:50 |
| Notch-3 | Proteintech | #551141-AP | 1:50 |
| Notch-4 | Abcam | #ab184742 | 1:50 |

**Supplementary Table 4. Antibodies used for Immunofluorescence in this study.**

| **Name** | **Company** | **Catalog Number** | **Concentration** |
| --- | --- | --- | --- |
| GFAP | Sigma | #SAB2500462 | 1:100 |
| MAP-2 | Proteintech | #67015-1-Ig | 1:100 |
| Thbs-2 | Abcam | #ab84469 | 1:100 |
| PSD-95 | Cell Signaling Technology | #3450 | 1:100 |
| Synaptotagmin | Abcam | #ab13259 | 1:100 |
| donkey anti-goat Alexa Fluor® 647 | Invitrogen | #A32849 | 1:100 |
| donkey anti-mouse Alexa Fluor® 555 | Invitrogen | #A31570 | 1:100 |
| donkey anti-rabbit Alexa Fluor® 488 | Invitrogen | #A21206 | 1:100 |
| goat anti-mouse IgG H&L (Alexa Fluor® 594) | Abcam | # ab150120 | 1:100 |
| goat anti-rabbit IgG H&L (Alexa Fluor® 488) | Abcam | # ab150077 | 1:100 |
