# Supplementary material for: Downregulation of microRNA-9-5p promotes synaptic remodeling in the chronic phase after traumatic brain injury
Source: Cell Death Dis. 2021 Jan 5;12(1):9. doi: 10.1038/s41419-020-03329-5 (PMC7790831; doi:10.1038/s41419-020-03329-5)
Supplement: Supplementary file 2 — Supplementary Figure legend [file 41419_2020_3329_MOESM2_ESM.docx]

**Supplementary Figure legends:**

**Supplementary Figure. 1 The alteration of miRNA-9-5p level treated with miRNA oligomers in the traumatic foci at 7 days after traumatic brain injury.**

(A)The alteration of miRNA-9-5p level treated with agomir in the traumatic foci after CCI. (B) The alteration of miRNA-9-5p level treated with antagomir in the traumatic foci after CCI. Error bars indicate mean ± SD. (*P<0.05, **P<0.01, ***P<0.001 versus the CON group; #P<0.05 versus the 50μM group; +P<0.05 versus the 100μM group) (n=5/group) (CCI: control cortex injury)

**Supplementary Figure. 2 The diffusion of miRNA-9-5p agomirs in cerebrospinal fluid in rats.**

(A)Coronal scan with white light. (B) Coronal scan with fluorescence microscope. (C)Sagittal scan with white light. (D) Sagittal scan with fluorescence microscope. (The arrow indicates the diffusion position)

**Supplementary Figure. 3 The alteration of miRNA-9-5p level treated with miRNA oligomers in the traumatic foci after traumatic brain injury.**

(A) The alteration of miRNA-9-5p level treated by single injection with agomirs and antagomirs in the traumatic foci. (B) The alteration of miRNA-9-5p level treated by double injection with agomirs and antagomirs in the traumatic foci. Error bars indicate mean ± SD. (*P<0.05, **P<0.01, ***P<0.001 versus the CCI group) (n=5/group) (CCI: control cortex injury)

**Supplementary Figure. 4** **The immunofluorescence stain around traumatic foci at 35 days after traumatic brain injury.**

The expression changing of Thbs-2 in neuron and astrocyte around traumatic foci was detected by immunofluorescence stain.

**Supplementary Figure. 5 Figure5. The schematic diagram of observation site for immunofluorescence stain.**

The observation site of cortex part was under the cortical lesion near to white matter as indicated by the white frame and arrow.

**Supplementary Figure. 6 The alteration of miRNA-9-5p level in the traumatic foci after traumatic brain injury.**

Figure6. The alteration of miRNA-9-5p level in the traumatic foci after traumatic brain injury.

The alteration of miRNA-9-5p level treated with agomirs and antagomirs in the traumatic foci after traumatic brain injury was detected by qRT-PCR. Error bars indicate mean ± SD. (△P<0.05, △△P<0.01, △△△P<0.001 versus the sham group) (n=5/group)
