# Supplementary figures and images for: Downregulation of microRNA-9-5p promotes synaptic remodeling in the chronic phase after traumatic brain injury
Source: Cell Death Dis. 2021 Jan 5;12(1):9. doi: 10.1038/s41419-020-03329-5 (PMC7790831; doi:10.1038/s41419-020-03329-5)

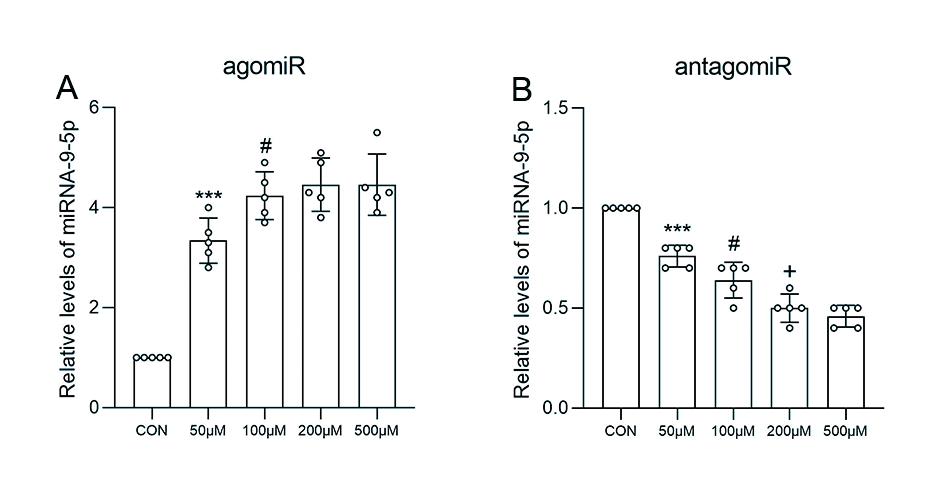

Supplement: Supplementary file 3 — supplement figure 1 [file 41419_2020_3329_MOESM3_ESM.tif]

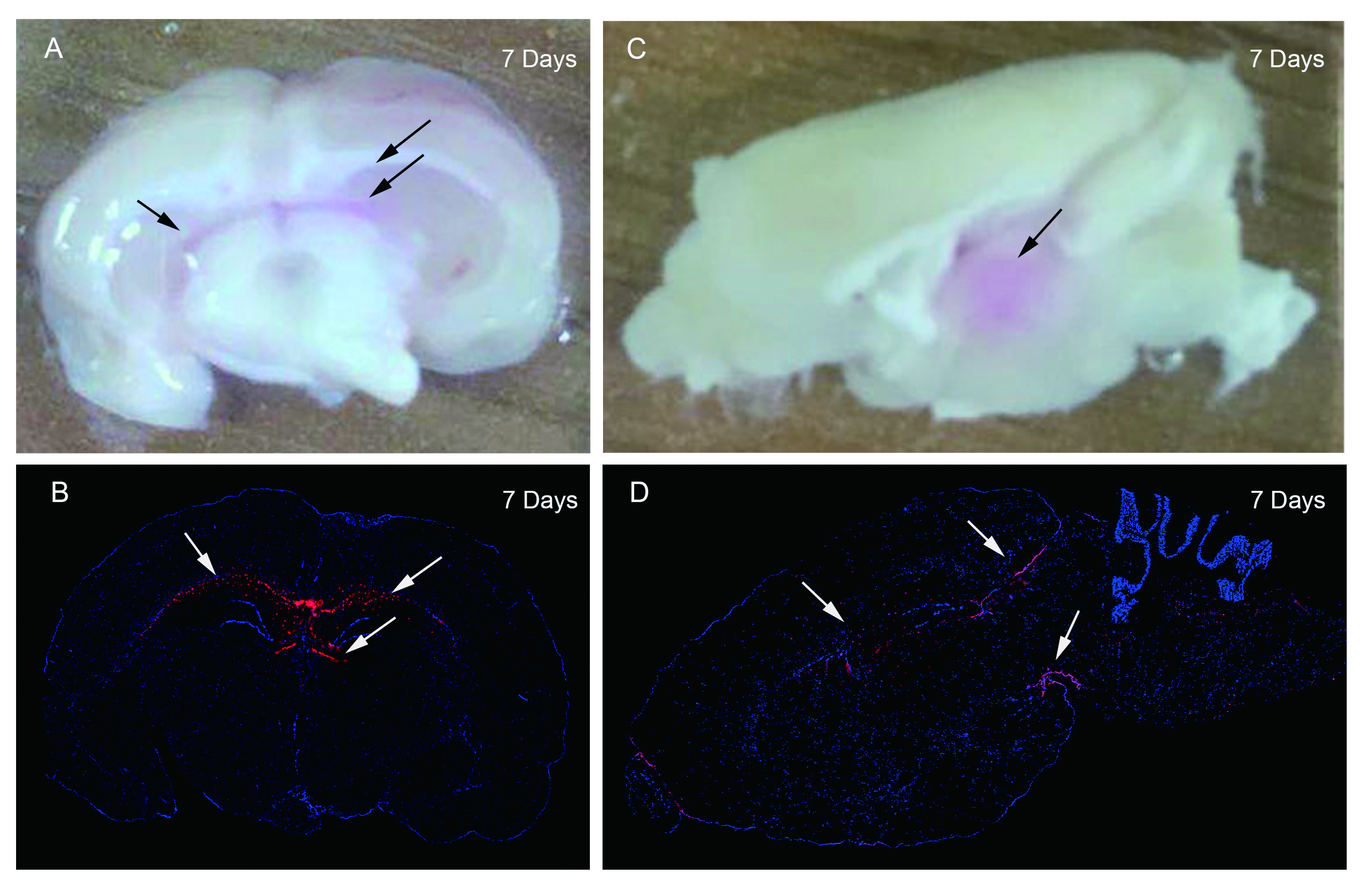

Supplement: Supplementary file 4 — supplement figure 2 [file 41419_2020_3329_MOESM4_ESM.tif]

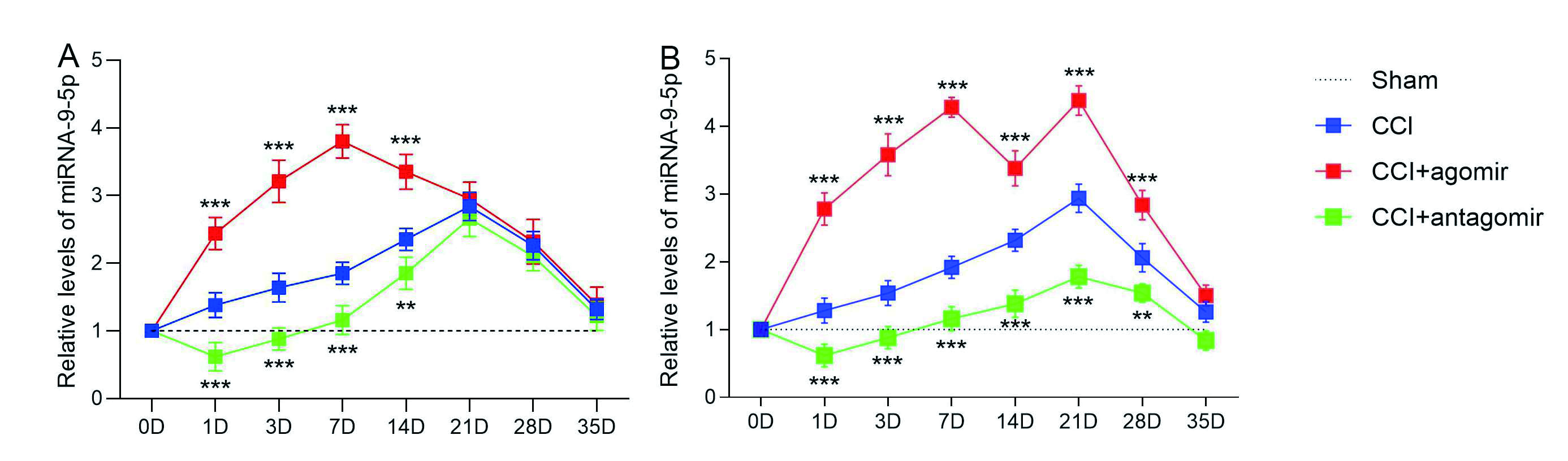

Supplement: Supplementary file 5 — supplement figure 3 [file 41419_2020_3329_MOESM5_ESM.tif]

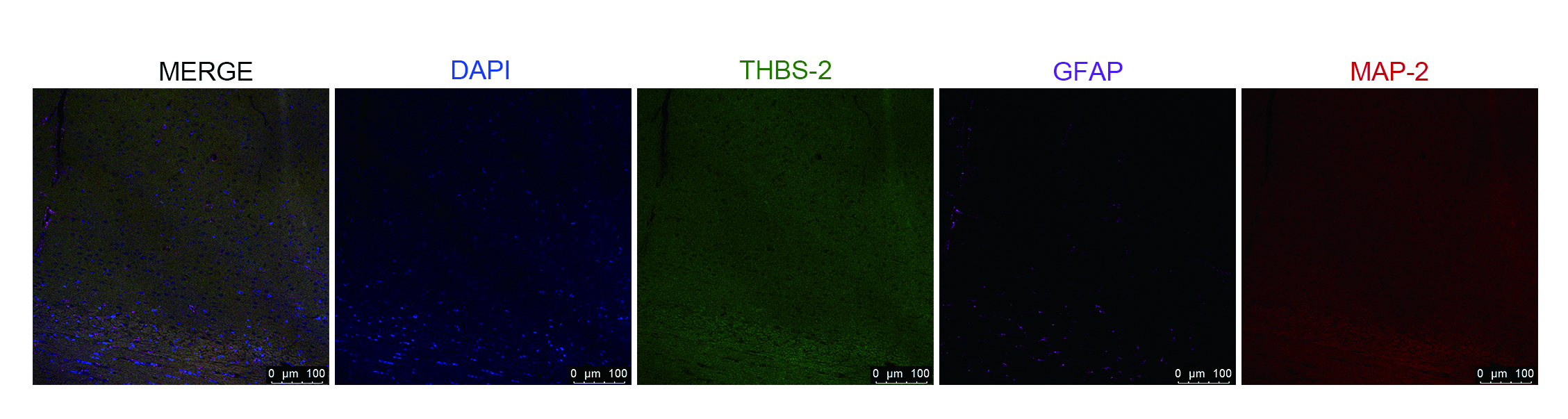

Supplement: Supplementary file 6 — supplement figure 4 [file 41419_2020_3329_MOESM6_ESM.tif]

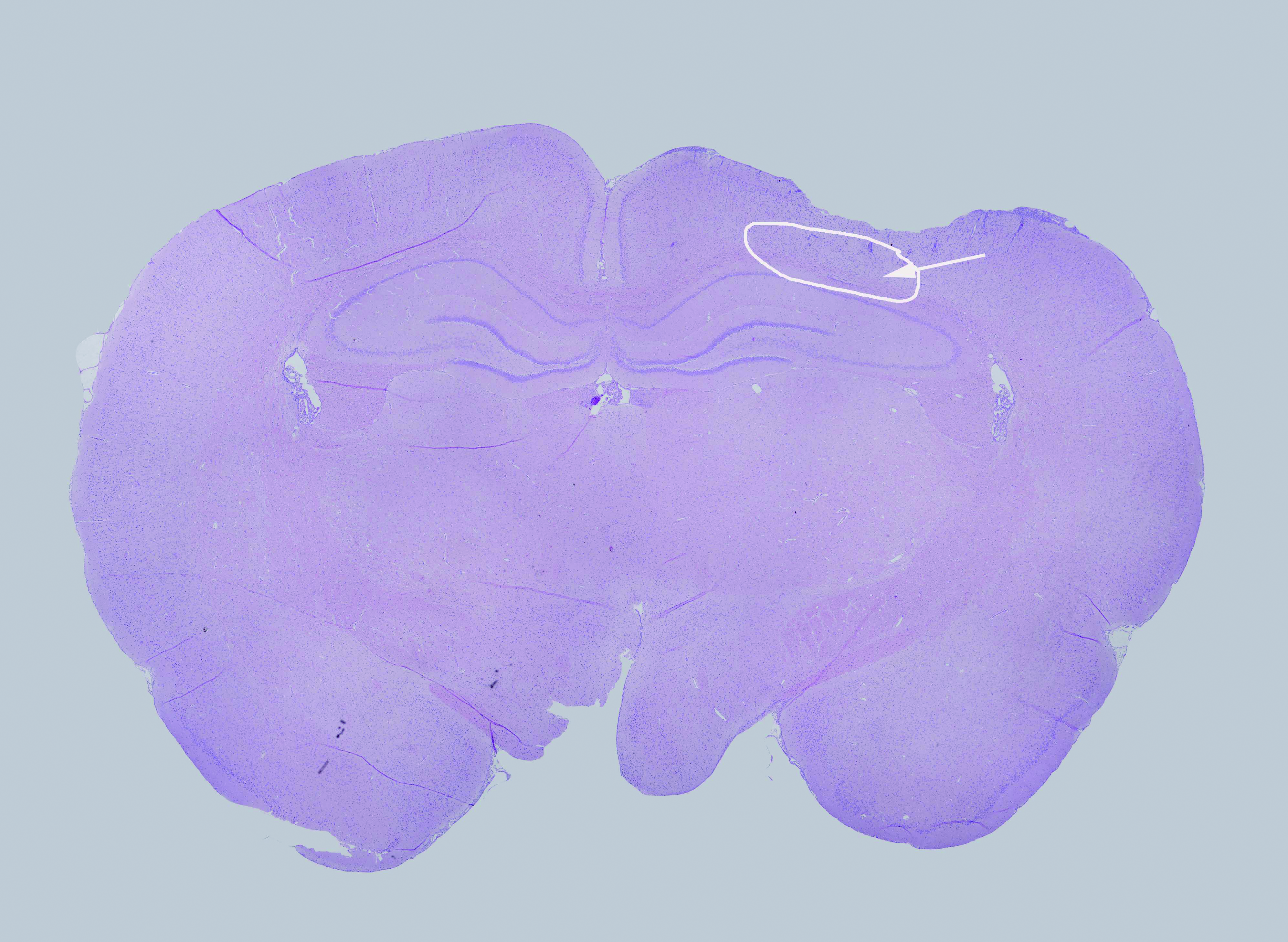

Supplement: Supplementary file 7 — supplement figure 5 [file 41419_2020_3329_MOESM7_ESM.tif]

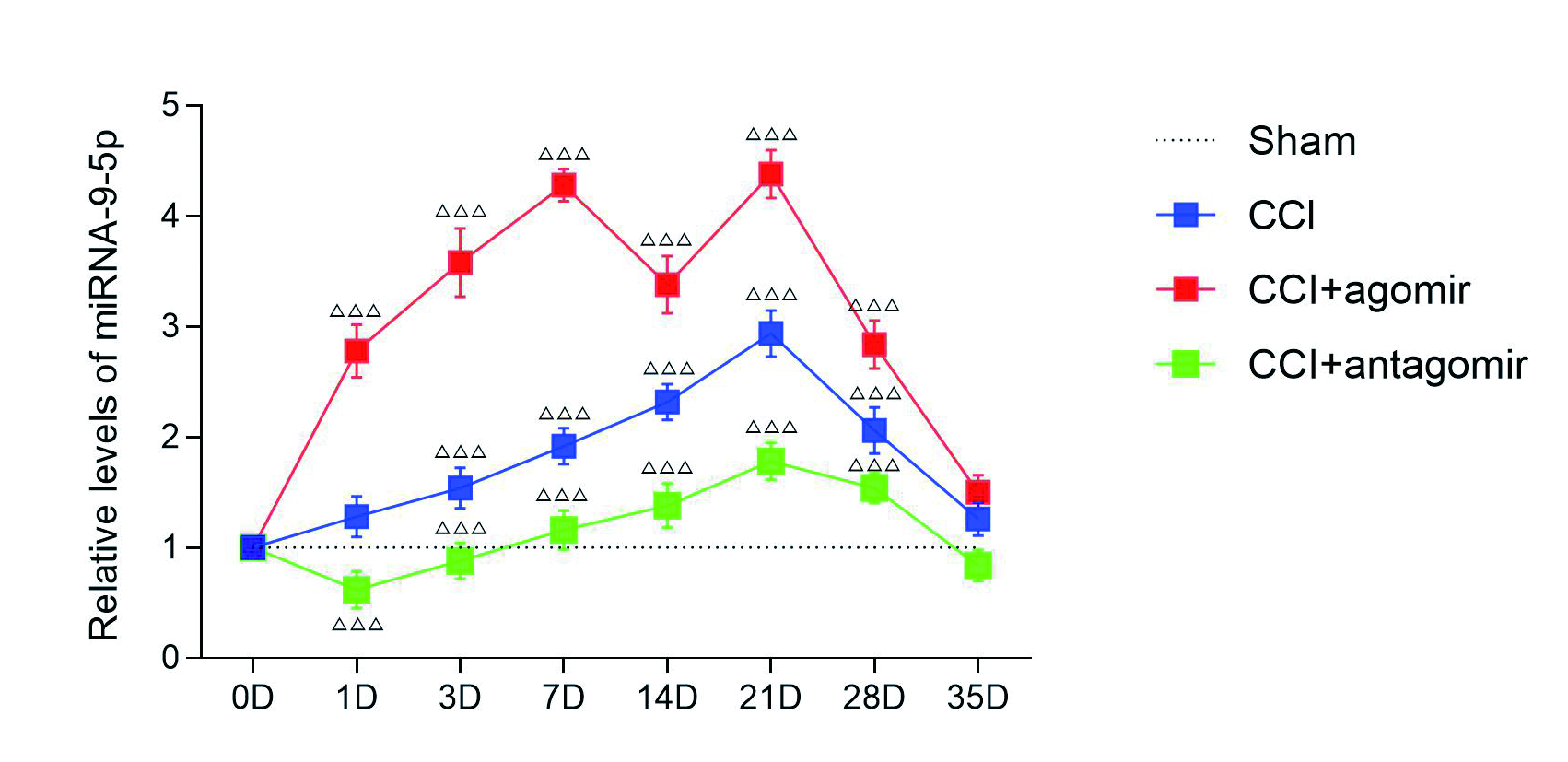

Supplement: Supplementary file 8 — supplement figure 6 [file 41419_2020_3329_MOESM8_ESM.tif]
